# Development and validation of a prediction model for iron status in a large U.S. cohort of women

**DOI:** 10.1038/s41598-023-42993-3

**Published:** 2023-10-12

**Authors:** Ann Von Holle, Katie M. O’Brien, Robert Janicek, Clarice R. Weinberg

**Affiliations:** 1 Biostatistics and Computational Biology Branch, National Institute of Environmental Health Sciences, Research Triangle Park, NC USA; 2 Epidemiology Branch, National Institute of Environmental Health Sciences, Research Triangle Park, NC USA; 3 Advanced Research and Diagnostic Laboratory, University of Minnesota, Minneapolis, MN USA; 4 Biostatistics and Computational Biology Branch, National Institute of Environmental Health Sciences, Mail Drop A3-03, P.O. Box 12233, Durham, NC 27709 USA

**Keywords:** Epidemiology, Predictive markers

## Abstract

Serum iron levels can be important contributors to health outcomes, but it is not often feasible to rely on blood-based measures for a large epidemiologic study. Predictive models that use questionnaire-based factors such as diet, supplement use, recency of blood donation, and medical conditions could potentially provide a noninvasive alternative for studying health effects associated with iron status. We hypothesized that a model based on questionnaire data could predict blood-based measures of iron status biomarkers. Using iron (mcg/dL), ferritin (mcg/dL), and transferrin saturation (%) based on blood collected at study entry, in a subsample from the U.S.-wide Sister Study (n = 3171), we developed and validated a prediction model for iron with multivariable linear regression models. Model performance based on these cross-sectional data was weak, with R^2^ less than 0.10 for serum iron and transferrin saturation, but better for ferritin, with an R^2^ of 0.13 in premenopausal women and 0.19 in postmenopausal women. When menopause was included in the predictive model for the sample, the R^2^ was 0.31 for ferritin. Internal validation of the estimates indicated some optimism present in the observed prediction model, implying there would be worse performance when applied to new samples from the same population. Serum iron status is hard to assess based only on questionnaire data. Reducing measurement error in both the exposure and outcome may improve the prediction model performance, but environmental heterogeneity, temporal variation, and genetic heterogeneity in absorption and storage may contribute substantially to iron status.

## Introduction

Known predictors of iron status in women include genetic factors, diet, supplement use, menopausal status and recency of menopause, recency of blood donation, and medical conditions. These predictors may together be usable as proxies for serum iron measures, which are not feasible to collect in large prospective studies, and which, when available in retrospective studies, are subject to bias from reverse causal effects.

Both dietary and non-dietary factors like blood loss can influence iron stores^1^. Dietary predictors include alcohol^2^ and heme iron intake from sources such as red meat^3,4^. There are numerous nondietary factors associated with iron levels, including: menstruation^5^, hormone use^6^, physical activity^7^, body mass index (BMI)^1,5^, iron supplements^1,5^, calcium supplements^8^, aspirin^1,9,10^, statins^11–14^, polycystic ovarian syndrome (PCOS)^15–17^, parity^18^, occult bleeding from a stomach ulcer^19^, previous diagnosis of iron deficiency anemia (IDA), and repetitive caesarean section^20^.

Recent studies also report blood donor status as a strong predictor of iron status in women^21,22^. Frequent and recent blood donation demonstrated the strongest association with low serum ferritin levels in a study of a Danish population from 2014^5^. In this study other factors associated with low serum ferritin levels included menopause status in women, weight, age, menstruation characteristics, supplements, and diet. This study underlines the importance of blood donor status and blood loss when considering iron status, with evidence of an association between iron deficiency anemia and blood donation.

Simultaneous assessment of the many common predictors of serum iron measures could contribute to existing knowledge and provide a chance to identify additional iron-related health outcomes through development of a predictive model. Such a model could enable use of common self-reported items from surveys as predictors to assess body iron status. Once the most jointly important predictors have been identified, one could use that set of predictors via a multiple imputation model to leverage a substudy based on a small subcohort that provides blood-based measures and scale up to an analysis of the entire cohort, including outcomes from non-subcohort members. Our primary aim is to develop and validate a prediction model of serum iron measures at baseline with many iron-related predictors related to absorption, metabolism and loss of iron, in a sample from a large U.S.-based cohort of women. We hypothesized that a prediction model based on questionnaire data in a cross-sectional study could serve as a proxy for blood-based measures to assess iron status. We used R^2^ as our main measure to assess our hypothesis that a questionnaire-based model performs well.

## Methods

### Study population

The original case-cohort study for this analysis contains 6008 participants with iron measures^23^ from the Sister Study^24^, a prospective cohort of 50,884 women who, at enrollment, had not been diagnosed with breast cancer themselves but had at least one sister diagnosed with breast cancer. Measures from the randomly selected subcohort, n = 3200 served as a random sample from the Sister Study. We omitted 29 participants missing all baseline serum iron measures, and among the 3171 remaining participants (Supplementary Fig. S1), we stratified the analyses by menopause status at baseline. We then modeled the sample as a whole, to estimate model performance for the entire sample with menopausal status as a predictor.

### Outcome variables for body iron levels

Our outcomes included: (1) serum iron (mcg/dL), (2) ferritin (mcg/dL), and (3) transferrin saturation (%). Serum sample handling and sample analysis have been previously described^23^. Briefly, a Roche Cobas 6000 Chemistry analyzer was used to perform analyses to measure serum iron, ferritin and unsaturated iron binding capacity (UIBC) with two levels of quality control and inter-assay coefficient of variation not exceeding 3.3%. Transferrin saturation was calculated as the ratio of serum iron to the sum of serum iron and UIBC, which ratio was then multiplied by 100 to create a percentage.

### Candidate predictors

We selected predictors of serum iron biomarkers based on individual associations previously reported in the literature. These predictors can be grouped into the following five categories: (1) supplements and dietary intake, (2) lifestyle, (3) reproductive history, (4) health conditions, and (5) a general category of binary predictors.

The first group included red meat consumption and supplements and dietary intake for calcium and iron, based on a nutrition analysis derived from a self-reported food frequency questionnaire that was conducted through a separate survey at baseline^24^. In addition to iron supplements (including multivitamins), we included calcium intake because calcium inhibits iron absorption^8^. The second group of continuous predictors, including lifestyle-related variables reported at study entry, included: exercise (hours per week), examiner-measured BMI (kg/m^2^), and alcohol consumption (drinks per month). The third group of variables included reproductive blood-loss related variables reported at baseline: years since last menstrual period, reproductive life span (calculated by subtracting age at menarche from age at most recent menstrual period), estrogen or progesterone use (number of total years), and total years pregnant or breastfeeding. The fourth group of variables included self-report of ever having the specified health condition at baseline: preeclampsia as a substitute for cesarean section (not available)^25^, polycystic ovary syndrome (PCOS), irritable bowel syndrome (IBS), colon/rectum polyps, and iron-deficiency anemia (IDA). A fifth, and final, group was composed of the following self-reported binary response variables: statin use, regular aspirin use (1 + /week for 3 or more months), and recent blood donation (within past 12 months).

All Sister Study participants provided written informed consent. The institutional review board of the National Institutes of Health, Bethesda, Maryland (United States of America), provides study approval and oversight (protocol number 02EN271). All methods for this study were carried out in accordance with relevant guidelines and regulations.

### Statistical analyses

The descriptive statistics included either the median and interquartile range for continuous variables or frequencies accompanied by percent distributions for categorical variables.

We estimated the association between individual predictors and serum iron outcomes by fitting simple linear regression models separately for each of the iron-related predictors, with age included in all models. The multivariable prediction model for iron status included all variables in one model for each of the three iron status markers. We also considered simpler predictive models based on a step-down procedure, with successive removal of predictors that were contributing little to the goodness of fit of the model (p > 0.05). We also considered a penalization method, the lasso model^26^, as a means to remove variables and simplify the predictor model since this model had the lowest mean squared error for the minimum alpha tuning parameter^27^ from the glmnet^28^ R package. To assess the performance and internal validation of these multivariable predictor models, we followed best practice guidelines for prediction models^29–31^, which are outlined below.

## Model performance and validation

To estimate model performance for the prediction models, we estimated the proportion explained variation, R^2^. Following best practices in prediction model reporting^29^, we conducted internal validation procedures for the prediction model to assess how the model would perform in new samples. Multivariable prediction models based on the observed data will typically be ‘optimistic,’ in that they provide better performance than would be found in new samples^32^, either from the same or different underlying populations. In our case, we estimated the performance of the prediction model compared to new samples drawn from the same underlying population using bootstrapping, using a method known as ‘internal validation’^32^. Finding similar data collected from a different population was not possible, and we did not conduct external validation measures.

For internal validation of performance measures, we used a repeated sampling approach^33^ with bootstrap samples, as defined by Harrell’s bias correction^34^. This method performs well and includes the following steps^34,35^: (1) estimate the prediction model and calculate performance statistics with the observed data, (2) generate a bootstrap sample with replacement from the original sample, (3) create a prediction model based on the bootstrap sample and generate the performance statistic (R^2^ and calibration slopes) – the ‘training’ estimate, (4) using the model parameter estimates from the bootstrap sample in step 3 and the observed data, calculate the performance statistic – the ‘testing’ estimate, (5) repeat steps 2–4 for each of the bootstrap samples. In our case we repeated steps 2–4 1000 times and calculated the ‘optimism’ of the original prediction model by averaging the difference between the performance statistics based on the fits to the bootstrap data and fits based on the original observed data (in steps 3 and 4). The ‘optimism’ estimates the bias indicating to what extent the performance statistic from the original model was too high relative to what would be seen with new samples from the same population.

In addition to R^2^, we also used calibration measures by pairing with the internal model validation described above to assess how well the prediction model based on the observed outcomes can function with new samples^30,32^. Calibration applies to many outcomes, including continuous variables^33^. Generally defined, the calibration model is derived by fitting a regression model with observed values as the outcome and predicted values as the covariate. In our internal validation analyses, the slope from a calibration model based on observed data and predictions based on the bootstrap-based parameter estimates, defined in step four above, served as an indicator of what amount the estimated parameters need to be changed to make the model fit well for predictions in new samples from the underlying population. A calibration slope of 1.0 indicates a well-calibrated fit, and a slope less than 1.0 suggests that the predictions are more extreme than the observed values and ‘overfitting’ the estimates^32^. This slope can also provide a uniform shrinkage factor to adjust coefficients for future use^32^.

## Missing data

None of the predictor variables had over 8 percent missing values for the premenopausal group or over 14 percent missing for the postmenopausal group. However, among the variables considered for the multivariable model, 17 percent (n = 181) of the premenopausal women and 24 percent (n = 498) of the postmenopausal women had at least one missing value. We used multiple imputation to account for the missing data in the multivariable models, assuming missing at random patterns. To estimate parameters using multiple imputation, we used the mice^36^ package in R to create imputed data sets. We also estimated the prediction models with only complete case data.

For the full multivariable model using the maximum likelihood method, we used 100 imputed samples to compute the estimates and pooled the estimates and standard errors according to Rubin’s method^37^. For the backwards selection models with the same set of covariates as for the full multivariable model, we used the psfmi^38^ package with Rubin’s Rules for variable selection^39–41^, in which the full model is fit in all imputed data sets, the estimates and standard errors are combined using Rubin’s Rules, the variable with the largest p-value is excluded, then this process is repeated until all parameters in the model have a p value below 0.05. To obtain imputed estimates for variable selection from the penalized regression models, we used the miselect^42^ package in R to implement a stacked pooled objective function across the imputed data sets^43^. For the model performance statistics, we estimated the statistic using the relevant model for each imputation, including bootstrapping, then averaged them.

We used R software^44^, version 3.6.1, to handle data and conduct all statistical analyses.

## Results

We excluded the supplemental breast cancer cases from the original case-cohort and only considered the 3171 randomly-sampled women in the subcohort, using only their data and blood sample taken at baseline. Of those women 2106 reported being postmenopausal and 1063 were premenopausal (Table 1). The median ferritin level was higher in the postmenopausal women. Spearman correlation values for the three iron outcome measures in the total sample (n = 3171) was 0.21 for serum iron and ferritin, 0.34 for transferrin saturation and ferritin, and 0.92 for serum iron and transferrin saturation.

**Table 1:** Baseline characteristics of subcohort by menopause status at baseline.

Variable	Total	Postmenopausal status at baseline in subcohort only	
		Yes	No
n	3171	2106	1063
Serum, baseline			
Iron, baseline (mcg/dL) (n = 3149) (median [IQR])	93.00 [74.00, 115.00]	93.00 [76.00, 114.00]	92.00 [69.00, 119.00]
Ferritin, baseline (mcg/dL) (n = 3153) (median [IQR])	67.00 [36.00, 112.00]	80.00 [49.00, 128.00]	40.00 [21.00, 75.00]
UIBC, baseline (n = 2987) (median [IQR])	231.00 [197.00, 269.00]	229.00 [197.00, 263.00]	235.50 [197.00, 285.00]
TIBC, baseline (n = 2986) (median [IQR])	328.00 [300.00, 360.00]	326.00 [299.00, 356.00]	332.00 [303.00, 369.00]
Transferrin saturation, baseline (%) (n = 2986) (median [IQR])	29.00 [22.00, 36.00]	29.00 [23.00, 36.00]	28.00 [20.00, 37.00]
Continuous			
Baseline age (years) (n = 3171) (median [IQR])	55.60 [48.90, 62.00]	59.50 [55.60, 65.20]	46.70 [43.10, 50.40]
BMI (kg/m\(^2\)) (n = 3168) (median [IQR])	26.52 [23.22, 31.13]	26.70 [23.47, 31.36]	25.91 [22.81, 30.64]
Age at menarche (years) (n = 3167) (median [IQR])	13.00 [12.00, 13.00]	13.00 [12.00, 13.00]	13.00 [12.00, 14.00]
Time since menopause (years) (n = 2106) (median [IQR])	9.00 [4.40, 14.90]	9.00 [4.40, 14.90]	0.00 [0.00, 0.00]
Age difference, serum draws (years) (n = 1227) (median [IQR])	7.60 [6.40, 8.70]	7.40 [6.30, 8.60]	8.00 [6.80, 8.80]
Number of pregnancies, excluding miscarriages and spontaneous abortions (n = 3168) (median [IQR])	2.00 [1.00, 3.00]	2.00 [1.00, 3.00]	2.00 [1.00, 3.00]
Total years pregnant and breastfeeding (n = 3151) (median [IQR])	2.65 [1.20, 6.21]	2.56 [1.42, 5.87]	2.94 [0.92, 6.95]
Iron, diet and supplements, mg (n = 3098) (median [IQR])	22.70 [12.50, 30.60]	24.60 [13.28, 31.30]	18.70 [11.10, 28.49]
Calcium, diet and supplements, mg (n = 3098) (median [IQR])	1304.95 [743.68, 1776.62]	1472.70 [853.55, 1858.75]	947.70 [622.80, 1536.50]
Categorical			
Smoking status (n = 3171) (%)			
Never	1746 (55.1)	1100 (52.2)	644 (60.6)
Past	1175 (37.1)	846 (40.2)	329 (31.0)
Current	250 (7.9)	160 (7.6)	90 (8.5)
Alcohol status (n = 2969) (%)			
Never	87 (2.9)	71 (3.6)	16 (1.6)
Past	623 (21.0)	458 (23.5)	164 (16.2)
Current	2259 (76.1)	1423 (72.9)	835 (82.3)
Education (n = 3171) (%)			
Less than high school degree	38 (1.2)	26 (1.2)	12 (1.1)
Completed high school or GED	479 (15.1)	369 (17.5)	110 (10.3)
Some college but no degree	612 (19.3)	411 (19.5)	201 (18.9)
Associate or technical degree	450 (14.2)	283 (13.4)	167 (15.7)
Bachelor’s degree	836 (26.4)	503 (23.9)	332 (31.2)
Doctoral or master’s degree	756 (23.8)	514 (24.4)	241 (22.7)
Age at first birth (years) (n = 3171) (%)			
Nulliparous	594 (18.7)	359 (17.0)	233 (21.9)
≤ 20	614 (19.4)	476 (22.6)	138 (13.0)
21–24	815 (25.7)	595 (28.3)	220 (20.7)
25–29	718 (22.6)	458 (21.7)	260 (24.5)
30 +	430 (13.6)	218 (10.4)	212 (19.9)
Race/ethnicity (n = 3171) (%)			
Non-Hispanic White	2654 (83.7)	1808 (85.8)	845 (79.5)
Non-Hispanic Black/African American	278 (8.8)	156 (7.4)	121 (11.4)
Hispanic/Latina	152 (4.8)	86 (4.1)	66 (6.2)
Other	87 (2.7)	56 (2.7)	31 (2.9)
BMI categories (kg/m^2^) (n = 3168) (%)			
< 18.5	29 (0.9)	23 (1.1)	6 (0.6)
18.5–24.9	1188 (37.5)	724 (34.4)	464 (43.7)
25.0–29.9	1006 (31.8)	709 (33.7)	296 (27.9)
30.0–34.9	548 (17.3)	380 (18.1)	167 (15.7)
35.0–39.9	244 (7.7)	172 (8.2)	72 (6.8)
40.0 +	153 (4.8)	97 (4.6)	56 (5.3)
Current statin user (n = 3107) (%)	783 (25.2)	645 (31.1)	138 (13.4)
Current regular aspirin user (n = 3106) (%)	913 (29.4)	742 (35.8)	171 (16.6)
Ever PCOS, yes/no (n = 3053) (%)	78 (2.6)	48 (2.4)	30 (2.9)
Ever preeclampsia, yes/no (n = 3163) (%)	188 (5.9)	109 (5.2)	79 (7.4)
Ever IBD, yes/no (n = 3129) (%)	45 (1.4)	34 (1.6)	11 (1.0)
Ever had colon/rectum polyps, yes/no (n = 2812) (%)	503 (17.9)	425 (23.2)	77 (7.9)
Blood donation in past 12 months, yes/no (n = 3166) (%)	442 (14.0)	267 (12.7)	174 (16.4)

### Univariable candidate predictor models

We characterized the associations between each of the predictors in the five categories and iron status in separate linear regression models. In the premenopausal group of women, strong positive associations with ferritin included red meat consumption, alcohol consumption, BMI, and current statin use (Supplementary Fig. S2, Supplementary Table S1). Strong inverse associations with ferritin were seen for years pregnant and breastfeeding, IDA, and recent blood donation. The associations with IDA and recent blood donation were also inverse for ferritin, transferrin saturation and serum iron. The association with BMI was positive for ferritin, but strongly inverse for transferrin saturation and serum iron. Red meat consumption, years pregnant or breastfeeding, and current statin use were only related to ferritin.

In the postmenopausal women, large positive associations with ferritin included: red meat consumption, alcohol consumption, and BMI (Supplementary Fig. S3, Supplementary Table S2). Strong inverse associations with ferritin existed for calcium intake, iron deficiency anemia (IDA), and recent blood donation. Some findings were different for the transferrin saturation and serum iron outcomes, including strong positive associations for exercise and inverse associations for BMI, years since last menstrual period (LMP), and statin use. In terms of consistency of direction of association across the three iron outcomes, alcohol consumption, recent blood donation and diagnosis with iron deficiency anemia all conformed to that pattern. Blood donation and IDA were both consistently strong predictors in both the pre- and postmenopausal groups.

### Model performance and validation

For the premenopausal group, after accounting for the missing values in the prediction models, we found that the estimated R^2^ for the multivariable prediction model was very low for both serum iron (0.07) and transferrin saturation (0.07) (Table 2). When conducting the analyses for the set of individuals with complete data, the R^2^ remains low but slightly higher for serum iron and transferrin saturation (Supplementary Table S3) compared to the prediction models accounting for the missing data. The multivariable prediction model for serum ferritin had a higher R^2^ (0.13), explaining more of the observed variation than the models for serum iron (R^2^ = 0.07) and transferrin saturation (R^2^ = 0.07). Internal validation of these estimates based on the bootstrap method suggests overfitting in the model, implying a reduced R^2^ after an optimism correction. The internal validation calibration slopes were between 0.9 and 1.0, suggesting that the predictions from models based on the observed data would tend to provide more extreme estimates when applied to new samples drawn from the same underlying population.

**Table 2:** Multivariable regression model parameter estimates by iron outcome for premenopausal group, multiple imputation.

Category	Variable label	ln (ferritin)			Serum iron			Transferrin saturation		
		Backwards selection	Lasso	ML estimate	Backwards selection	Lasso	ML estimate (95% CI)	Backwards selection	Lasso	ML estimate
				(95% CI)						(95% CI)
Age	Age (years)	0.019		0.014 (0.003, 0.026)			− 0.005 (− 0.017, 0.008)			− 0.002 (− 0.015, 0.010)
Binary predictors	Currently use aspirin regularly (1 + /wk, 3 + mos)		0.037	0.098 (− 0.064, 0.261)			0.018 (− 0.151, 0.186)			0.019 (− 0.155, 0.193)
	Currently use statins		0.073	0.140 (− 0.037, 0.317)			− 0.008 (− 0.188, 0.173)			− 0.042 (− 0.231, 0.146)
	Donate blood in past 12 months, y/n	− 0.694	− 0.546	− 0.700 (− 0.854, − 0.546)	− 0.249	− 0.124	− 0.253 (− 0.412, − 0.094)	− 0.391	− 0.267	− 0.394 (− 0.555, − 0.232)
Health conditions (ever)	Ever dx with IDA	− 0.32	− 0.208	− 0.347 (− 0.486, − 0.208)	− 0.184	− 0.074	− 0.175 (− 0.322, − 0.029)	− 0.205	− 0.094	− 0.214 (− 0.362, − 0.066)
	Ever had colon/rectum polyps			0.016 (− 0.208, 0.241)			− 0.069 (− 0.296, 0.157)			0.010 (− 0.222, 0.242)
	Ever had irritable bowel disease, y/n		0.036	0.592 (0.036, 1.148)			0.038 (− 0.539, 0.614)			0.124 (− 0.449, 0.698)
	Ever had PCOS, y/n			0.112 (− 0.233, 0.457)			− 0.207 (− 0.560, 0.145)			− 0.181 (− 0.546, 0.184)
	Ever had preeclampsia, y/n	0.426	0.196	0.388 ( 0.168, 0.608)			0.018 (− 0.207, 0.244)			0.089 (− 0.139, 0.317)
Lifestyle	Alcohol (drinks per month)	0.008	0.006	0.008 (0.005, 0.011)	0.006	0.004	0.006 (0.003, 0.009)	0.004	0.002	0.004 (0.001, 0.007)
	BMI kg/m2		0.003	0.008 (− 0.001, 0.017)	− 0.031	− 0.026	− 0.030 (− 0.040, − 0.021)	− 0.032	− 0.027	− 0.033 (− 0.043, − 0.023)
	Exercise hours per month			0.003 (− 0.002, 0.009)	0.007	0.004	0.007 (0.001, 0.012)	0.006	0.002	0.005 (− 0.001, 0.011)
Reproductive health	Years pregnant and breastfeeding	− 0.01	− 0.002	− 0.009 (− 0.018, 0.000)			0.001 (− 0.009, 0.010)			− 0.004 (− 0.014, 0.006)
Supplement and Diet	Dietary + supplement calcium, dg			− 0.003 (− 0.014, 0.008)		0.001	0.007 (− 0.005, 0.018)		< 0.001	0.004 (− 0.007, 0.016)
	Dietary + supplement iron, mg			0.002 (− 0.002, 0.006)			0.000 (− 0.004, 0.004)			0.001 (− 0.003, 0.006)
	Meat (1 oz. eq per week)	0.018	0.009	0.016 (0.006, 0.025)			0.004 (− 0.006, 0.014)			0.003 (− 0.007, 0.013)
Performance	Apparent R^2^			0.134			0.065			0.071
	Calibration slope			0.924			0.907			0.928
	Harrell corrected R^2^			0.109			0.045			0.054

In the postmenopausal group of women, after accounting for the missing values, we found that the estimated R^2^ for the predictor model was low for both serum iron (0.06) and transferrin saturation (0.09) (Table 3). The model predicting serum ferritin measures had a higher R^2^ (0.19), explaining more of the observed variation than the models for serum iron and transferrin saturation. When conducting the analyses restricting to the set of individuals with complete data, the R^2^ remained low but was slightly higher for serum iron and transferrin saturation (Supplementary Table S4) relative to the prediction models accounting for missing data. Internal validation of these estimates based on bootstraps suggests overfitting in the model implying a reduced optimism-corrected R^2^. Similar to the premenopausal group of women, the internal validation calibration slopes remain between 0.9 and 1.0, suggesting that the model would provide somewhat reduced strength of associations between the predictors and the outcomes in new samples drawn from the same underlying population.

**Table 3:** Multivariable regression model parameter estimates by iron outcome for postmenopausal group, multiple imputation.

Category	Variable label	ln (ferritin)			Serum iron			Transferrin saturation		
		Backwards selection	Lasso	ML estimate (95% CI)	Backwards selection	Lasso	ML estimate (95% CI)	Backwards selection	Lasso	ML estimate (95% CI)
Age	Age (years)	0.012		0.013 (0.004, 0.022)			0.003 (− 0.007, 0.013)	0.009		0.010 (0.000, 0.020)
Binary predictors	Currently use aspirin regularly (1 + /wk, 3 + mos)			0.056 (− 0.028, 0.140)	0.108	0.01	0.125 (0.035, 0.216)	0.127	0.007	0.134 (0.043, 0.226)
	Currently use statins			0.024 (− 0.063, 0.110)			− 0.081 (− 0.173, 0.012)	− 0.119		− 0.119 (− 0.213, − 0.025)
	Donate blood in past 12 months, y/n	− 1.118	− 0.92	− 1.121 (− 1.238, − 1.004)	− 0.167	− 0.03	− 0.167 (− 0.292, − 0.041)	− 0.412	− 0.27	− 0.406 (− 0.533, − 0.279)
Health conditions (ever)	Ever dx with IDA	− 0.233	− 0.06	− 0.234 (− 0.330, − 0.138)	− 0.107	− 0.01	− 0.101 (− 0.204, 0.002)	− 0.118	− 0.01	− 0.105 (− 0.209, − 0.001)
	Ever had colon/rectum polyps			− 0.046 (− 0.148, 0.055)			− 0.067 (− 0.175, 0.041)			− 0.067 (− 0.176, 0.043)
	Ever irritable bowel disease, y/n			0.151 (− 0.159, 0.461)			0.260 (− 0.074, 0.593)			0.240 (− 0.094, 0.575)
	Ever had PCOS, y/n			− 0.056 (− 0.319, 0.206)			− 0.024 (− 0.304, 0.256)			− 0.133 (− 0.409, 0.142)
	Ever had preeclampsia, y/n			0.107 (− 0.068, 0.283)			− 0.015 (− 0.203, 0.173)			− 0.003 (− 0.191, 0.185)
Lifestyle	Alcohol (drinks per month)	0.005	0.001	0.006 (0.004, 0.008)	0.004	0.002	0.004 (0.002, 0.006)	0.003	0.001	0.002 (0.000, 0.005)
	BMI kg/m2			0.003 (− 0.003, 0.010)	− 0.034	− 0.03	− 0.033 (− 0.040, − 0.025)	− 0.038	− 0.03	− 0.037 (− 0.044, − 0.029)
	Exercise hours per month			0.000 (− 0.004, 0.003)	0.004	0.001	0.004 (0.000, 0.007)			0.003 (0.000, 0.007)
Reproductive health	Estrogen or progesterone use (years)			− 0.005 (− 0.011, 0.001)			0.007 (0.000, 0.013)			0.004 (− 0.002, 0.011)
	Reproductive life span (years)			− 0.003 (− 0.011, 0.004)			0.002 (− 0.006, 0.010)			0.000 (− 0.009, 0.008)
	Years pregnant and breastfeeding			− 0.001 (− 0.008, 0.007)			0.001 (− 0.007, 0.009)			− 0.002 (− 0.010, 0.007)
	Years since LMP at interview			0.001 (− 0.005, 0.008)			− 0.007 (− 0.014, 0.000)	− 0.006		− 0.008 (− 0.016, − 0.001)
Supplement and diet	Dietary + supplement calcium, dg	− 0.013		− 0.014 (− 0.021, − 0.007)			0.003 (− 0.004, 0.011)			− 0.004 (− 0.011, 0.004)
	Dietary + supplement iron, mg			0.001 (− 0.002, 0.004)			− 0.002 (− 0.005, 0.001)			− 0.001 (− 0.004, 0.002)
	Meat (1 oz. eq per week)	0.019	0.006	0.018 (0.012, 0.024)			0.006 (− 0.001, 0.012)	0.009	0.001	0.010 (0.003, 0.017)
Performance	Apparent R^2^			0.189			0.064			0.086
	Calibration slope			0.979			0.918			0.927
	Harrell corrected R^2^			0.178			0.05			0.07

We find similar patterns in performance when combining the pre- and postmenopausal groups of women in one model (Supplementary Table S5). The model with a ferritin outcome has the highest R^2^ (0.31) compared to serum iron (0.08) and transferrin saturation (0.09). Because menopause status is predictive in the combined sample, the amount of explained variation is greater than in the strata based on the two menopausal subgroups. Models explaining more of the variance, in this case indicated by R^2^, are better performing prediction models. Models with an R^2^ approaching 100% imply the observed outcomes are very close to the predicted values, and a value near 0% implies no association between the observed and predicted values^32^. The value of R^2^ near 0.31 implies 69% of the variance remains unexplained.

## Discussion

In this work, we evaluated prediction models for iron status using questionnaire-based data, including many variables relating to diet, supplements, lifestyle-related variables, reproductive characteristics, iron-related health conditions, medications, and recent blood donation status. We developed this model with a large sample of women in the United States, ages 35–74 years, with three different common biomarkers for iron status based on serum: iron, transferrin saturation, and ferritin. Although each person in that sample had a sister previously diagnosed with breast cancer, we know of no reason to expect that history to affect the relationship between questionnaire variables and their iron status.

Model performance was weak, with an R^2^ less than 0.10 for the maximum likelihood models for iron and transferrin saturation but stronger, with an R^2^ of 0.13 in the premenopausal group and 0.19 in the postmenopausal group for ferritin. The overall R^2^ for ferritin with both menopausal groups in the same model was 0.31. The internal validation calibration slopes were close to 1, showing good calibration between observed and predicted outcomes, for all models in the internal validation process. Internal validation calibration slopes greater than 0.9 suggest a tendency of the observed prediction model to provide similar predictions in new samples from the underlying population. Using the variable selection approaches, including backwards selection and penalized regression, did not appear to substantively change the estimated model performance measures in the complete case sample. We did not assess predictions of dichotomous outcomes, such as iron deficiency or overload based on a cutoff value for ferritin, but such a model may be worth considering in future work.

The weak model performance does not support the use of a large array of predictive variables to replace common serum measures. The predictive model for serum ferritin demonstrated the best performance, especially for the postmenopausal group of women, with an R^2^ close to 0.20. No menopause-stratified prediction model R^2^ exceeded 0.20, indicating that within each of the menopause categories over 80% of the variance remained unexplained. When we combined pre- and post-menopausal groups into one analysis (Supplementary Table S5), the explained variance (R^2^) for the serum ferritin outcome was 0.31. In this model menopause was a strong predictor and consequently stratifying by menopause reduces the explained variance. However, even that model leaves around 69% of the variance unexplained. Nevertheless, the calibration slope from the internal validation was close to one, 0.98, for the ferritin outcome in the postmenopausal group, indicating that this model should provide predicted values in new samples from the same underlying population that are not systematically different from those observed.

Although this study used easily accessible self-reported variables, collecting information for all candidate predictions in these analyses may not be possible when resources are limited. Based on the variable reduction models, including stepwise backwards selection and lasso models, we identified several variables, such as recent blood donation, that remained consistently in the model and explained a sizable amount of the observed variance, although the total fraction explained was low. Recent blood donation was the predictor with the strongest association with iron outcomes for both pre- and postmenopausal groups of women, and it remained in the model with both variable selection approaches. This finding is consistent with results from a European sample of blood donors^21,22^. We considered only cross-sectional data and investigators instead carrying out longitudinal analyses could presumably capture important temporal variation in iron status by acquiring careful records of the timing of repeated donations of whole blood.

There could also be reverse-causal effects when cross-sectional data are used for assessing iron status. For example, a history of IDA, heavy menstruation or regular blood donation could prompt some to take regular iron supplements. Although not as strong an association as the blood donation indicator variable, lifestyle measures frequently remained after variable selection. Alcohol and red meat consumption remained in both pre- and postmenopausal groups after variable selection. The indicator for ever having preeclampsia was unexpectedly positively associated with ferritin and remained in the model for the premenopausal group only, providing a more informative predictor for this group than for the postmenopausal group. This may be in part a birth cohort or recall bias effect since the pregnancies tend to be farther in the past for the post-menopause group. The basis for that association is not clear, but it is possible, as mentioned above, that the positive association with ferritin may reflect an effect of inflammation^45^ on ferritin and not actual iron status. There is, however, evidence that iron overload plays a causative role in preeclampsia^46^.

### Strengths and limitations

One strength in assessing prediction models of iron status was the evaluation of three commonly measured serum-based iron biomarkers and a comprehensive set of predictors that can be easily obtained through questionnaires – and, excepting food frequency questionnaires, readily incorporated into electronic health systems – related to body iron status. Moreover, we assessed these variables in a large random sample from a contemporary U.S. cohort of women. From this information, we assessed the suitability of using questionnaire items to assess iron status through a multivariable prediction model. These strengths of this study had the potential to show, provided we found evidence of strong prediction model performance, a means to extend analyses based on serum iron measures to larger samples through a proxy prediction model based on more accessible measures.

Unfortunately, none of the predictive models showed strong performance, indicating the possibility that the variation among women in these markers of iron status depends on additional variables outside of our candidates. Measurement error could be impacting both the predictor and outcome variables, which would affect model performance. Also, questionnaire items, no matter how extensive, are inherently error-prone, given the potential for misclassification or recall bias. Another limitation is the known temporal variability of the outcome variables, especially for serum iron and transferrin saturation^47^.

An inherent limitation relates to the role of inflammation, which we did not have a good measure for, and which is known to elevate ferritin levels and disrupt its ability to serve as a marker for iron status^48^. Some of our questionnaire data could have acted as markers for chronic inflamation, e.g. IBD, and also the use of statins, aspirin, high BMI, history of preeclampsia, all of which are related to heart disease and therefore inflammation and all of which predicted higher ferritin but not higher serum iron or percent transferrin saturation (see supplemental figures).

Another limitation of this study is the inability to carry out external validation–and inability to generalize this prediction model to other populations, such as men. This study population was women ages 35–74 years who had at least one sister diagnosed with breast cancer at study entry and was disproportionately non-Hispanic White. The extensive nature of this study, given the large number of predictor variables paired with serum iron values, prevented us from finding a similar sample in a different population and conducting external validation analyses. External validation presents a future area of research with the potential of a similar prediction model providing better performance in different populations such as men.

In developing prediction models based on easily accessible questionnaire data, we did not find evidence to support that questionnaire-based prediction models could serve as a good proxy for serum iron measures. When developing and validating the prediction models, we determined that serum ferritin served as a more predictable outcome than either serum iron or transferrin saturation. The prediction models for the postmenopausal group of women had better performance measures than those for the premenopausal group. Although more extensive and precise predictor variables may improve the performance of the model, it seems likely that unmeasured genetic and environmental factors may cause temporal variability and heterogeneity among women in their iron status. It was also of note that recency of blood donation was the most important factor for any group of women and any outcome measured, underlining the consistency and importance of collecting a detailed history of blood donation recency and frequency in determining iron status. Creating predictive models for body iron status based on questionnaire data that could usefully replace the iron markers measured in serum samples remains a challenge.

### Supplementary Information

Supplementary Information.

## Data Availability

The datasets generated and/or analyzed during the current study are available from the corresponding author on reasonable request (https://sisterstudy.niehs.nih.gov/).
