# Supplementary material for: Development and validation of a prediction model for iron status in a large U.S. cohort of women
Source: Sci Rep. 2023 Oct 12;13:17309. doi: 10.1038/s41598-023-42993-3 (PMC10570329; doi:10.1038/s41598-023-42993-3)
Supplement: Supplementary file 1 — Supplementary Information. [file 41598_2023_42993_MOESM1_ESM.docx]

Title:

Development and validation of a prediction model for iron status in a large U.S. cohort of women

Authors:

Von Holle, Ann^a^

^a^ Biostatistics and Computational Biology Branch National Institute of Environmental Health Sciences, Research Triangle Park, NC, United States

O’Brien, Katie M.^b^

^b^ Epidemiology Branch, National Institute of Environmental Health Sciences, Research Triangle Park, NC, United States

Janicek, Robert^c^

^c^ Advanced Research and Diagnostic Laboratory, University of Minnesota, Minneapolis, MN, United States

Weinberg, Clarice R.^a^

^a^ Biostatistics and Computational Biology Branch, National Institute of Environmental Health Sciences, Research Triangle Park, NC, United States

Corresponding Author Information:

Dr. Clarice R. Weinberg

P.O. Box 12233

Mail Drop A3-03

Durham, N.C. 27709

Tel. 984-287-3697

[weinberg@niehs.nih.gov](mailto:weinberg@niehs.nih.gov)


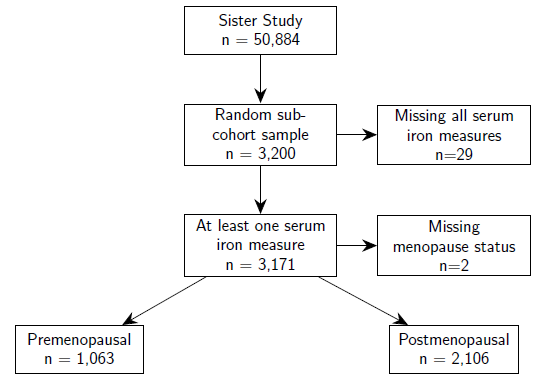


Supplementary Figure 1. Participant Flow Diagram. Flow diagram shows the inclusion criteria for the participants of the Sister Study Sample at baseline, 2003-2009.


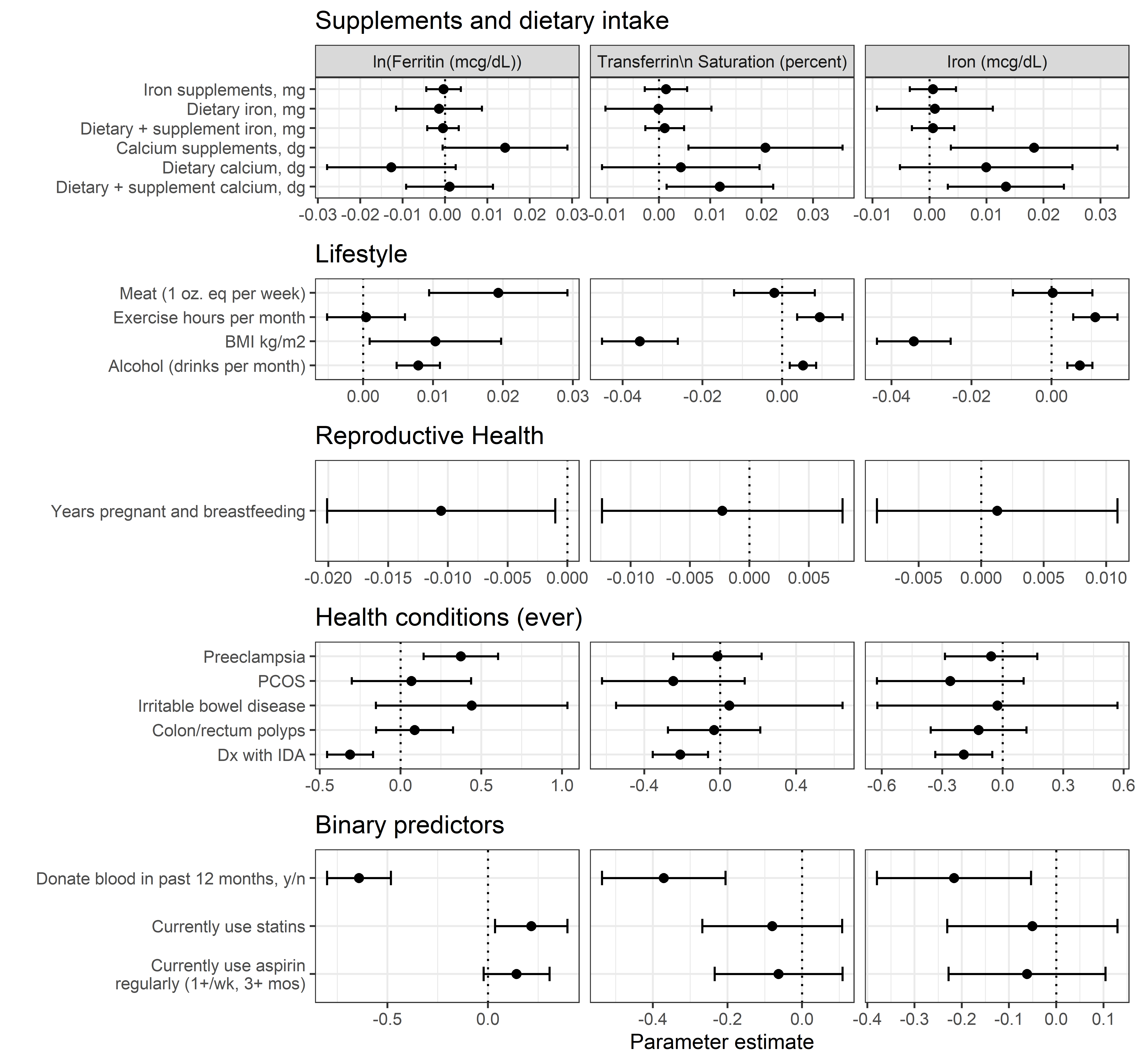


Supplementary Figure 2. Univariate regression model coefficients for candidate predictors for premenopausal women. Each estimate represents a separate age-adjusted coefficient for each predictor, represented in the rows of the figures, of the serum iron outcomes, represented in the columns of the figures. Coefficient values indicate the change in the serum iron outcome for a one unit increase in the predictor. Coefficient values exceeding zero represent a positive association between the predictor and the iron outcome and values below zero indicate inverse associations. The bars represent 95 percent confidence intervals for the coefficient. Strong positive associations with ferritin included red meat consumption, alcohol consumption, BMI, and current statin. Strong inverse associations with ferritin were seen for years pregnant and breastfeeding, IDA, and recent blood donation. Abbreviations: BMI, body mass index; Dx, diagnosis; IDA, iron deficiency anemia; PCOS, polycystic ovarian syndrome; wk, week; mos, months; oz, ounce.


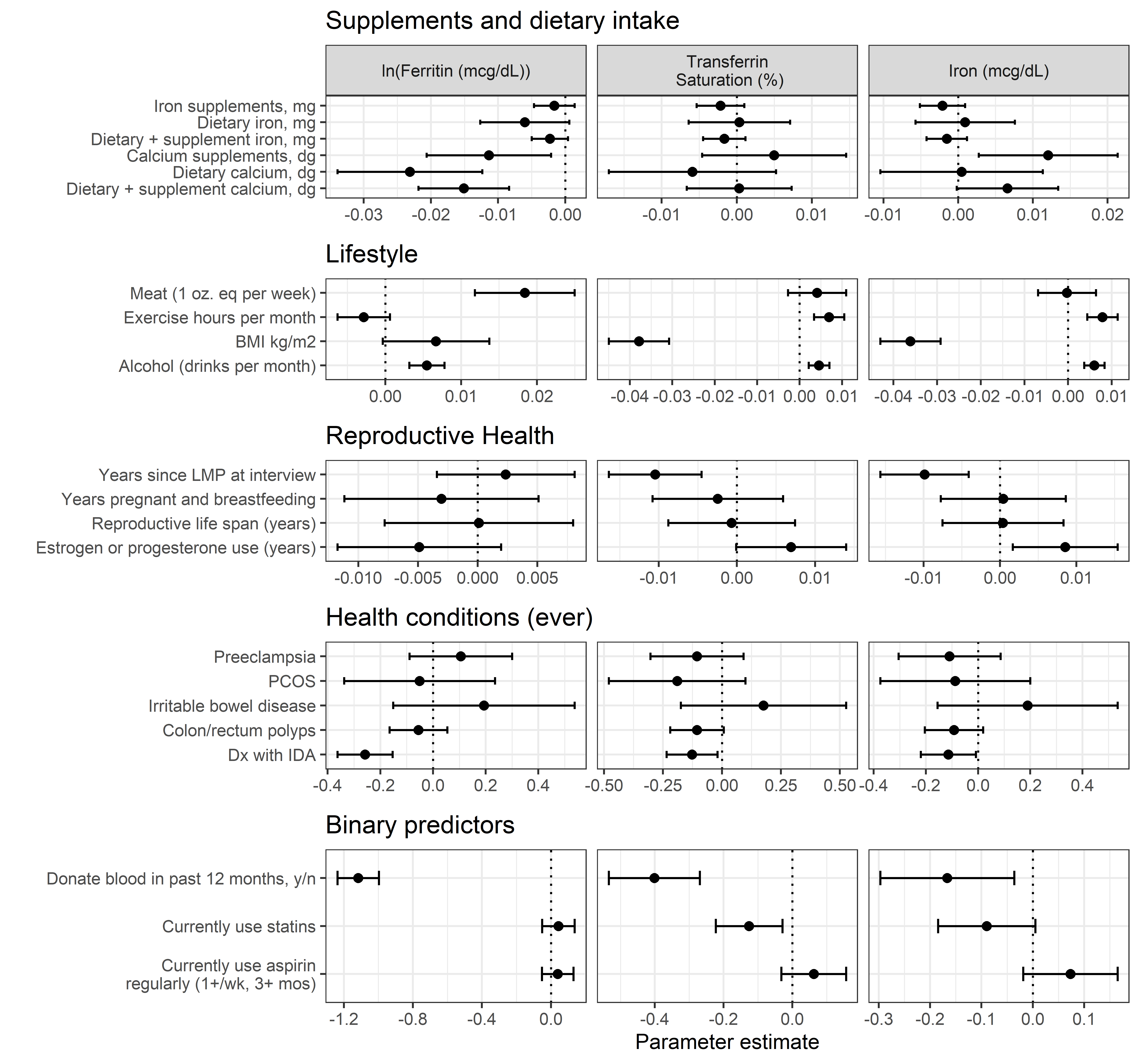


Supplementary Figure 3. Univariate regression model coefficients for candidate predictors for postmenopausal women. Each estimate represents a separate age-adjusted coefficient for each predictor, represented in the rows of the figures, of the serum iron outcomes, represented in the columns of the figures. Coefficient values indicate the change in the serum iron outcome for a one unit increase in the predictor. Coefficient values exceeding zero represent a positive association between the predictor and the iron outcome and values below zero indicate inverse associations. The bars represent 95 percent confidence intervals for the coefficient. Large positive associations with ferritin included: red meat consumption, alcohol consumption, and BMI. Strong inverse associations with ferritin existed for calcium intake, iron deficiency anemia, and recent blood donation. Abbreviations: Dx, diagnosis; IDA, iron deficiency anemia; PCOS, polycystic ovarian syndrome; wk, week; mos, months; oz, ounce.

Supplementary materials

Contents

[Supplementary Table S1. Univariate regression model coefficients for candidate predictors for premenopausal women 7](#_Toc121476890)

[Supplementary Table S2. Univariate regression model coefficients for candidate predictors for postmenopausal women 12](#_Toc121476891)

[Supplementary Table S3. Multivariable regression model parameter estimates by iron outcome for premenopausal group, complete cases 18](#_Toc121476892)

[Supplementary Table S4. Multivariable regression model parameter estimates by iron outcome for postmenopausal group, complete cases 20](#_Toc121476893)

[Supplementary Table S5. Multivariable regression model parameter estimates by iron outcome for combined group of pre- and postmenopausal group, complete cases 22](#_Toc121476894)

# Supplementary Table S1. Univariate regression model coefficients for candidate predictors for premenopausal women

| **Category** | **Variable** | **Outcome** | **Estimate (95% CI)** |
| --- | --- | --- | --- |
| Binary predictors | Currently use aspirin regularly (1+/wk, 3+ mos) | ln(Ferritin) | 0.142, (-0.022, 0.306) |
|  | Currently use aspirin regularly (1+/wk, 3+ mos) | Transferrin saturation | -0.063, (-0.235, 0.108) |
|  | Currently use aspirin regularly (1+/wk, 3+ mos) | Serum iron | -0.062, (-0.228, 0.104) |
|  | Currently use statins | ln(Ferritin) | 0.215, (0.035, 0.396) |
|  | Currently use statins | Transferrin saturation | -0.08, (-0.267, 0.107) |
|  | Currently use statins | Serum iron | -0.051, (-0.231, 0.129) |
|  | Donate blood in past 12 months, y/n | ln(Ferritin) | -0.642, (-0.801, -0.483) |
|  | Donate blood in past 12 months, y/n | Transferrin saturation | -0.371, (-0.537, -0.205) |
|  | Donate blood in past 12 months, y/n | Serum iron | -0.217, (-0.38, -0.054) |
| Health conditions (ever) | Ever dx with IDA | ln(Ferritin) | -0.313, (-0.455, -0.17) |
|  | Ever dx with IDA | Transferrin saturation | -0.21, (-0.356, -0.064) |
|  | Ever dx with IDA | Serum iron | -0.194, (-0.335, -0.052) |
|  | Ever had colon/rectum polyps | ln(Ferritin) | 0.087, (-0.151, 0.325) |
|  | Ever had colon/rectum polyps | Transferrin saturation | -0.032, (-0.276, 0.211) |
|  | Ever had colon/rectum polyps | Serum iron | -0.12, (-0.357, 0.117) |
|  | Ever irritable bowel disease , y/n | ln(Ferritin) | 0.44, (-0.153, 1.034) |
|  | Ever irritable bowel disease , y/n | Transferrin saturation | 0.047, (-0.549, 0.644) |
|  | Ever irritable bowel disease , y/n | Serum iron | -0.027, (-0.622, 0.569) |
|  | Ever PCOS , y/n | ln(Ferritin) | 0.067, (-0.303, 0.436) |
|  | Ever PCOS , y/n | Transferrin saturation | -0.247, (-0.623, 0.128) |
|  | Ever PCOS , y/n | Serum iron | -0.26, (-0.624, 0.103) |
|  | Ever preeclampsia , y/n | ln(Ferritin) | 0.373, (0.142, 0.603) |
|  | Ever preeclampsia , y/n | Transferrin saturation | -0.015, (-0.248, 0.218) |
|  | Ever preeclampsia , y/n | Serum iron | -0.058, (-0.287, 0.172) |
| Lifestyle | Alcohol (drinks per month) | ln(Ferritin) | 0.008, (0.005, 0.011) |
|  | Alcohol (drinks per month) | Transferrin saturation | 0.005, (0.002, 0.009) |
|  | Alcohol (drinks per month) | Serum iron | 0.007, (0.004, 0.01) |
|  | BMI kg/m2 | ln(Ferritin) | 0.01, (0.001, 0.02) |
|  | BMI kg/m2 | Transferrin saturation | -0.036, (-0.045, -0.026) |
|  | BMI kg/m2 | Serum iron | -0.034, (-0.044, -0.025) |
|  | Exercise hours per month | ln(Ferritin) | 0, (-0.005, 0.006) |
|  | Exercise hours per month | Transferrin saturation | 0.009, (0.004, 0.015) |
|  | Exercise hours per month | Serum iron | 0.011, (0.005, 0.017) |
|  | Meat (1 oz. eq per week) | ln(Ferritin) | 0.019, (0.009, 0.029) |
|  | Meat (1 oz. eq per week) | Transferrin saturation | -0.002, (-0.012, 0.008) |
|  | Meat (1 oz. eq per week) | Serum iron | 0, (-0.01, 0.01) |
| Reproductive health | Years pregnant and breastfeeding | ln(Ferritin) | -0.011, (-0.02, -0.001) |
|  | Years pregnant and breastfeeding | Transferrin saturation | -0.002, (-0.012, 0.008) |
|  | Years pregnant and breastfeeding | Serum iron | 0.001, (-0.008, 0.011) |
| Supplement and Diet | Calcium supplements, dg | ln(Ferritin) | 0.014, (-0.001, 0.029) |
|  | Calcium supplements, dg | Transferrin saturation | 0.021, (0.006, 0.036) |
|  | Calcium supplements, dg | Serum iron | 0.018, (0.004, 0.033) |
|  | Dietary + supplement calcium, dg | ln(Ferritin) | 0.001, (-0.009, 0.011) |
|  | Dietary + supplement calcium, dg | Transferrin saturation | 0.012, (0.001, 0.022) |
|  | Dietary + supplement calcium, dg | Serum iron | 0.013, (0.003, 0.024) |
|  | Dietary + supplement iron, mg | ln(Ferritin) | 0, (-0.004, 0.003) |
|  | Dietary + supplement iron, mg | Transferrin saturation | 0.001, (-0.003, 0.005) |
|  | Dietary + supplement iron, mg | Serum iron | 0.001, (-0.003, 0.004) |
|  | Dietary calcium, dg | ln(Ferritin) | -0.013, (-0.028, 0.003) |
|  | Dietary calcium, dg | Transferrin saturation | 0.004, (-0.011, 0.02) |
|  | Dietary calcium, dg | Serum iron | 0.01, (-0.005, 0.025) |
|  | Dietary iron, mg | ln(Ferritin) | -0.001, (-0.012, 0.009) |
|  | Dietary iron, mg | ln(Ferritin) | -0.001, (-0.012, 0.009) |
|  | Dietary iron, mg | Transferrin saturation | 0, (-0.01, 0.01) |
|  | Dietary iron, mg | Transferrin saturation | 0, (-0.01, 0.01) |
|  | Dietary iron, mg | Serum iron | 0.001, (-0.009, 0.011) |
|  | Dietary iron, mg | Serum iron | 0.001, (-0.009, 0.011) |
|  | Iron supplements, mg | ln(Ferritin) | 0, (-0.004, 0.004) |
|  | Iron supplements, mg | Transferrin saturation | 0.001, (-0.003, 0.005) |
|  | Iron supplements, mg | Serum iron | 0.001, (-0.003, 0.005) |

Note: Each row represents a separate regression model and all models are adjusted for age at study entry.

# Supplementary Table S2. Univariate regression model coefficients for candidate predictors for postmenopausal women

| **Category** | **Variable** | **Outcome** | **Estimate (95% CI)** |
| --- | --- | --- | --- |
| Binary predictors | Currently use aspirin regularly (1+/wk, 3+ mos) | ln(Ferritin) | 0.038, (-0.053, 0.129) |
|  | Currently use aspirin regularly (1+/wk, 3+ mos) | Transferrin saturation | 0.062, (-0.032, 0.156) |
|  | Currently use aspirin regularly (1+/wk, 3+ mos) | Serum iron | 0.073, (-0.019, 0.165) |
|  | Currently use statins | ln(Ferritin) | 0.043, (-0.052, 0.137) |
|  | Currently use statins | Transferrin saturation | -0.126, (-0.223, -0.029) |
|  | Currently use statins | Serum iron | -0.09, (-0.184, 0.005) |
|  | Donate blood in past 12 months, y/n | ln(Ferritin) | -1.117, (-1.237, -0.997) |
|  | Donate blood in past 12 months, y/n | Transferrin saturation | -0.402, (-0.534, -0.269) |
|  | Donate blood in past 12 months, y/n | Serum iron | -0.167, (-0.297, -0.036) |
| Health conditions (ever) | Ever dx with IDA | ln(Ferritin) | -0.258, (-0.363, -0.153) |
|  | Ever dx with IDA | Transferrin saturation | -0.127, (-0.235, -0.019) |
|  | Ever dx with IDA | Serum iron | -0.114, (-0.22, -0.009) |
|  | Ever had colon/rectum polyps | ln(Ferritin) | -0.056, (-0.165, 0.054) |
|  | Ever had colon/rectum polyps | Transferrin saturation | -0.106, (-0.219, 0.008) |
|  | Ever had colon/rectum polyps | Serum iron | -0.092, (-0.204, 0.019) |
|  | Ever irritable bowel disease , y/n | ln(Ferritin) | 0.193, (-0.151, 0.538) |
|  | Ever irritable bowel disease , y/n | Transferrin saturation | 0.176, (-0.174, 0.527) |
|  | Ever irritable bowel disease , y/n | Serum iron | 0.19, (-0.155, 0.535) |
|  | Ever PCOS , y/n | ln(Ferritin) | -0.051, (-0.338, 0.235) |
|  | Ever PCOS , y/n | Transferrin saturation | -0.19, (-0.48, 0.099) |
|  | Ever PCOS , y/n | Serum iron | -0.088, (-0.375, 0.2) |
|  | Ever preeclampsia , y/n | ln(Ferritin) | 0.105, (-0.089, 0.3) |
|  | Ever preeclampsia , y/n | Transferrin saturation | -0.106, (-0.304, 0.092) |
|  | Ever preeclampsia , y/n | Serum iron | -0.109, (-0.305, 0.086) |
| Lifestyle | Alcohol (drinks per month) | ln(Ferritin) | 0.005, (0.003, 0.008) |
|  | Alcohol (drinks per month) | Transferrin saturation | 0.005, (0.002, 0.007) |
|  | Alcohol (drinks per month) | Serum iron | 0.006, (0.004, 0.008) |
|  | BMI kg/m2 | ln(Ferritin) | 0.007, (0, 0.014) |
|  | BMI kg/m2 | Transferrin saturation | -0.038, (-0.045, -0.031) |
|  | BMI kg/m2 | Serum iron | -0.036, (-0.043, -0.029) |
|  | Exercise hours per month | ln(Ferritin) | -0.003, (-0.006, 0.001) |
|  | Exercise hours per month | Transferrin saturation | 0.007, (0.003, 0.011) |
|  | Exercise hours per month | Serum iron | 0.008, (0.004, 0.011) |
|  | Meat (1 oz. eq per week) | ln(Ferritin) | 0.018, (0.012, 0.025) |
|  | Meat (1 oz. eq per week) | Transferrin saturation | 0.004, (-0.003, 0.011) |
|  | Meat (1 oz. eq per week) | Serum iron | 0, (-0.007, 0.006) |
| Reproductive health | Estrogen or progesterone use (years) | ln(Ferritin) | -0.005, (-0.012, 0.002) |
|  | Estrogen or progesterone use (years) | Transferrin saturation | 0.007, (0, 0.014) |
|  | Estrogen or progesterone use (years) | Serum iron | 0.009, (0.002, 0.015) |
|  | Reproductive life span (years) | ln(Ferritin) | 0, (-0.008, 0.008) |
|  | Reproductive life span (years) | Transferrin saturation | -0.001, (-0.009, 0.007) |
|  | Reproductive life span (years) | Serum iron | 0, (-0.008, 0.008) |
|  | Years pregnant and breastfeeding | ln(Ferritin) | -0.003, (-0.011, 0.005) |
|  | Years pregnant and breastfeeding | Transferrin saturation | -0.002, (-0.011, 0.006) |
|  | Years pregnant and breastfeeding | Serum iron | 0, (-0.008, 0.009) |
|  | Years since LMP at interview | ln(Ferritin) | 0.002, (-0.003, 0.008) |
|  | Years since LMP at interview | Transferrin saturation | -0.01, (-0.016, -0.005) |
|  | Years since LMP at interview | Serum iron | -0.01, (-0.016, -0.004) |
| Supplement and Diet | Calcium supplements, dg | ln(Ferritin) | -0.011, (-0.021, -0.002) |
|  | Calcium supplements, dg | Transferrin saturation | 0.005, (-0.005, 0.015) |
|  | Calcium supplements, dg | Serum iron | 0.012, (0.003, 0.021) |
|  | Dietary + supplement calcium, dg | ln(Ferritin) | -0.015, (-0.022, -0.008) |
|  | Dietary + supplement calcium, dg | Transferrin saturation | 0, (-0.007, 0.007) |
|  | Dietary + supplement calcium, dg | Serum iron | 0.007, (0, 0.013) |
|  | Dietary + supplement iron, mg | ln(Ferritin) | -0.002, (-0.005, 0) |
|  | Dietary + supplement iron, mg | Transferrin saturation | -0.002, (-0.004, 0.001) |
|  | Dietary + supplement iron, mg | Serum iron | -0.002, (-0.004, 0.001) |
|  | Dietary calcium, dg | ln(Ferritin) | -0.023, (-0.034, -0.012) |
|  | Dietary calcium, dg | Transferrin saturation | -0.006, (-0.017, 0.005) |
|  | Dietary calcium, dg | Serum iron | 0, (-0.01, 0.011) |
|  | Dietary iron, mg | ln(Ferritin) | -0.006, (-0.013, 0.001) |
|  | Dietary iron, mg | ln(Ferritin) | -0.006, (-0.013, 0.001) |
|  | Dietary iron, mg | Transferrin saturation | 0, (-0.006, 0.007) |
|  | Dietary iron, mg | Transferrin saturation | 0, (-0.006, 0.007) |
|  | Dietary iron, mg | Serum iron | 0.001, (-0.006, 0.008) |
|  | Dietary iron, mg | Serum iron | 0.001, (-0.006, 0.008) |
|  | Iron supplements, mg | ln(Ferritin) | -0.002, (-0.005, 0.001) |
|  | Iron supplements, mg | Transferrin saturation | -0.002, (-0.005, 0.001) |
|  | Iron supplements, mg | Serum iron | -0.002, (-0.005, 0.001) |

Note: Each row represents a separate regression model and all models are adjusted for age at study entry and years since last menstrual period.

# Supplementary Table S3. Multivariable regression model parameter estimates by iron outcome for premenopausal group, complete cases

|  | | | **ln(Ferritin)** | | | | **Serum Iron** | | | | **Transferrin Saturation** | | |
| --- | --- | --- | --- | --- | --- | --- | --- | --- | --- | --- | --- | --- | --- |
| **Category** | **Variable** | **ML estimate (95% CI)** | | **Backwards selection** | **Lasso** | **ML estimate (95% CI)** | | **Backwards selection** | **Lasso** | **ML estimate (95% CI)** | | **Backwards selection** | **Lasso** |
| Age | Age (years) | 0.019 ( 0.006, 0.032) | | 0.022 | 0.017 | -0.002 (-0.015, 0.012) | |  |  | 0.002 (-0.012, 0.015) | |  |  |
| Binary predictors | Currently use aspirin regularly (1+/wk, 3+ mos) | 0.145 (-0.031, 0.320) | |  | 0.098 | 0.079 (-0.099, 0.257) | |  |  | 0.083 (-0.101, 0.267) | |  |  |
|  | Currently use statins | 0.093 (-0.100, 0.285) | |  | 0.071 | -0.064 (-0.257, 0.129) | |  |  | -0.104 (-0.304, 0.097) | |  |  |
|  | Donate blood in past 12 months, y/n | -0.679 (-0.852, -0.507) | | -0.679 | -0.605 | -0.283 (-0.456, -0.110) | | -0.273 | -0.221 | -0.453 (-0.629, -0.277) | | -0.428 | -0.364 |
| Health conditions (ever) | Ever dx with IDA | -0.357 (-0.514, -0.201) | | -0.345 | -0.301 | -0.144 (-0.301, 0.012) | |  | -0.083 | -0.186 (-0.346, -0.027) | |  | -0.105 |
|  | Ever had colon/rectum polyps | 0.024 (-0.219, 0.267) | |  |  | -0.070 (-0.313, 0.174) | |  |  | 0.024 (-0.226, 0.273) | |  |  |
|  | Ever irritable bowel disease , y/n | 0.544 (-0.120, 1.209) | |  | 0.310 | -0.013 (-0.686, 0.659) | |  |  | 0.189 (-0.481, 0.860) | |  |  |
|  | Ever PCOS , y/n | 0.105 (-0.274, 0.484) | |  |  | -0.471 (-0.848, -0.094) | |  | -0.303 | -0.397 (-0.781, -0.014) | |  | -0.199 |
|  | Ever preeclampsia , y/n | 0.355 ( 0.105, 0.604) | | 0.364 | 0.290 | -0.068 (-0.317, 0.181) | |  |  | -0.006 (-0.257, 0.245) | |  |  |
| Lifestyle | Alcohol (drinks per month) | 0.008 ( 0.004, 0.011) | | 0.008 | 0.007 | 0.005 ( 0.002, 0.009) | | 0.006 | 0.004 | 0.004 ( 0.001, 0.008) | |  | 0.003 |
|  | BMI kg/m2 | 0.005 (-0.005, 0.016) | |  | 0.003 | -0.034 (-0.045, -0.024) | | -0.037 | -0.032 | -0.035 (-0.046, -0.024) | | -0.038 | -0.033 |
|  | Exercise hours per month | 0.002 (-0.004, 0.009) | |  |  | 0.007 ( 0.001, 0.013) | |  | 0.004 | 0.005 (-0.001, 0.011) | |  | 0.003 |
|  | Meat (1 oz. eq per week) | 0.014 ( 0.003, 0.024) | |  | 0.011 | 0.004 (-0.006, 0.015) | |  | 0.001 | 0.003 (-0.008, 0.014) | |  |  |
| Reproductive health | Years pregnant and breastfeeding | -0.010 (-0.021, 0.000) | |  | -0.009 | -0.001 (-0.012, 0.009) | |  | 0.000 | -0.006 (-0.017, 0.005) | |  | -0.002 |
| Supplement and Diet | Dietary + supplement calcium, dg | -0.004 (-0.016, 0.008) | |  |  | 0.009 (-0.004, 0.021) | |  | 0.002 | 0.007 (-0.005, 0.019) | |  | 0.004 |
|  | Dietary + supplement iron, mg | 0.000 (-0.004, 0.005) | |  |  | -0.002 (-0.006, 0.003) | |  |  | 0.000 (-0.004, 0.005) | |  |  |
| Performance | Apparent R^2^ | 0.160 | | 0.120 | 0.142 | 0.102 | | 0.081 | 0.093 | 0.134 | | 0.084 | 0.108 |
|  | Calibration slope | 0.867 | |  |  | 0.867 | |  |  | 0.867 | |  |  |
|  | Harrell corrected R^2^ | 0.109 | | 0.092 | 0.126 | 0.067 | | 0.057 | 0.089 | 0.076 | | 0.062 | 0.102 |

# Supplementary Table S4. Multivariable regression model parameter estimates by iron outcome for postmenopausal group, complete cases

|  | | | **ln(Ferritin)** | | | | **Serum Iron** | | | | **Transferrin Saturation** | | |
| --- | --- | --- | --- | --- | --- | --- | --- | --- | --- | --- | --- | --- | --- |
| **Category** | **Variable** | **ML estimate**  **(95% CI)** | | **Backwards selection** | **Lasso** | **ML estimate**  **(95% CI)** | | **Backwards selection** | **Lasso** | **ML estimate**  **(95% CI)** | | **Backwards selection** | **Lasso** |
|  | Age (years) | 0.011 ( 0.001, 0.021) | |  | 0.008 | 0.003 (-0.008, 0.014) | |  |  | 0.008 (-0.003, 0.019) | |  |  |
| Binary predictors | Currently use aspirin regularly (1+/wk, 3+ mos) | 0.086 (-0.010, 0.181) | |  | 0.071 | 0.135 ( 0.031, 0.239) | |  | 0.095 | 0.156 ( 0.052, 0.260) | | 0.145 | 0.098 |
|  | Currently use statins | 0.040 (-0.058, 0.139) | |  | 0.029 | -0.075 (-0.182, 0.033) | |  | -0.020 | -0.115 (-0.222, -0.008) | |  | -0.065 |
|  | Donate blood in past 12 months, y/n | -1.128 (-1.262, -0.994) | | -1.123 | -1.093 | -0.224 (-0.369, -0.078) | |  | -0.156 | -0.440 (-0.585, -0.294) | | -0.435 | -0.373 |
| Health conditions (ever) | Ever dx with IDA | -0.183 (-0.295, -0.071) | |  | -0.160 | -0.086 (-0.207, 0.036) | |  | -0.017 | -0.071 (-0.192, 0.050) | |  | -0.013 |
|  | Ever had colon/rectum polyps | -0.057 (-0.166, 0.052) | |  | -0.045 | -0.075 (-0.194, 0.043) | |  | -0.020 | -0.098 (-0.216, 0.020) | |  | -0.039 |
|  | Ever irritable bowel disease , y/n | 0.062 (-0.301, 0.425) | |  |  | 0.175 (-0.219, 0.570) | |  |  | 0.117 (-0.274, 0.508) | |  |  |
|  | Ever PCOS , y/n | -0.060 (-0.351, 0.232) | |  |  | 0.038 (-0.279, 0.354) | |  |  | -0.039 (-0.351, 0.273) | |  |  |
|  | Ever preeclampsia , y/n | 0.167 (-0.035, 0.370) | |  | 0.137 | 0.037 (-0.183, 0.258) | |  |  | 0.030 (-0.186, 0.246) | |  |  |
| Lifestyle | Alcohol (drinks per month) | 0.005 ( 0.003, 0.008) | | 0.005 | 0.005 | 0.004 ( 0.001, 0.007) | | 0.004 | 0.002 | 0.003 ( 0.000, 0.005) | |  | 0.002 |
|  | BMI kg/m2 | 0.000 (-0.008, 0.008) | |  |  | -0.034 (-0.043, -0.026) | | -0.036 | -0.034 | -0.039 (-0.048, -0.031) | | -0.042 | -0.037 |
|  | Exercise hours per month | 0.000 (-0.004, 0.004) | |  |  | 0.003 (-0.001, 0.008) | |  | 0.001 | 0.003 (-0.001, 0.007) | |  | 0.001 |
|  | Meat (1 oz. eq per week) | 0.018 ( 0.011, 0.025) | | 0.019 | 0.017 | 0.005 (-0.003, 0.012) | |  | 0.001 | 0.010 ( 0.003, 0.018) | |  | 0.006 |
| Reproductive health | Estrogen or progesterone use (years) | -0.006 (-0.014, 0.001) | |  | -0.003 | 0.004 (-0.004, 0.012) | |  |  | 0.004 (-0.005, 0.012) | |  |  |
|  | Reproductive life span (years) | -0.002 (-0.011, 0.007) | |  |  | 0.005 (-0.005, 0.014) | |  | 0.001 | 0.003 (-0.007, 0.012) | |  | 0.003 |
|  | Years pregnant and breastfeeding | -0.002 (-0.011, 0.006) | |  |  | 0.001 (-0.008, 0.010) | |  |  | -0.001 (-0.010, 0.008) | |  |  |
|  | Years since LMP at interview | 0.005 (-0.003, 0.013) | |  | 0.005 | -0.006 (-0.014, 0.002) | |  | -0.001 | -0.007 (-0.015, 0.002) | |  | 0.000 |
| Supplement and Diet | Dietary + supplement calcium, dg | -0.014 (-0.022, -0.007) | | -0.013 | -0.011 | 0.004 (-0.004, 0.013) | |  |  | -0.003 (-0.012, 0.005) | |  |  |
|  | Dietary + supplement iron, mg | 0.002 (-0.001, 0.005) | |  | 0.001 | -0.002 (-0.005, 0.001) | |  |  | -0.001 (-0.004, 0.002) | |  |  |
| Performance | Apparent R^2^ | 0.210 | | 0.182 | 0.191 | 0.078 | | 0.059 | 0.064 | 0.110 | | 0.087 | 0.098 |
|  | Calibration slope | 0.882 | |  |  | 0.882 | |  |  | 0.882 | |  |  |
|  | Harrell corrected R^2^ | 0.178 | | 0.168 | 0.183 | 0.055 | | 0.044 | 0.063 | 0.074 | | 0.070 | 0.096 |

Note: Each row represents a separate regression model and all models are adjusted for age at study entry.

# Supplementary Table S5. Multivariable regression model parameter estimates by iron outcome for combined group of pre- and postmenopausal group, complete cases

|  | | **ln(Ferritin)** | | | **Serum Iron** | | | **Transferrin Saturation** | | |
| --- | --- | --- | --- | --- | --- | --- | --- | --- | --- | --- |
| **Category** | **Variable** | **ML estimate (95% CI)** | **Backwards selection** | **Lasso** | **ML estimate (95% CI)** | **Backwards selection** | **Lasso** | **ML estimate (95% CI)** | **Backwards selection** | **Lasso** |
| Age | Age (years) | 0.005 (-0.002, 0.012) |  | 0.004 | 0.000 (-0.008, 0.007) |  |  | 0.003 (-0.005, 0.010) |  | 0.000 |
| Binary predictors | Currently use aspirin regularly (1+/wk, 3+ mos) | 0.087 ( 0.007, 0.166) |  | 0.073 | 0.112 ( 0.022, 0.201) |  | 0.071 | 0.125 ( 0.034, 0.216) | 0.130 | 0.085 |
|  | Currently use statins | 0.043 (-0.040, 0.126) |  | 0.039 | -0.063 (-0.157, 0.031) |  | -0.011 | -0.105 (-0.200, -0.011) |  | -0.051 |
|  | Donate blood in past 12 months, y/n | -0.860 (-0.960, -0.761) | -0.860 | -0.821 | -0.259 (-0.371, -0.148) | -0.253 | -0.211 | -0.453 (-0.565, -0.341) | -0.452 | -0.403 |
| Health conditions (ever) | Ever dx with IDA | -0.227 (-0.313, -0.142) | -0.223 | -0.203 | -0.104 (-0.200, -0.008) |  | -0.054 | -0.111 (-0.207, -0.015) |  | -0.066 |
|  | Ever had colon/rectum polyps | -0.046 (-0.140, 0.048) |  | -0.033 | -0.071 (-0.177, 0.035) |  | -0.009 | -0.070 (-0.177, 0.037) |  | -0.009 |
|  | Ever irritable bowel disease , y/n | 0.135 (-0.165, 0.436) |  | 0.030 | 0.129 (-0.209, 0.468) |  |  | 0.129 (-0.209, 0.467) |  |  |
|  | Ever PCOS , y/n | 0.016 (-0.200, 0.232) |  |  | -0.205 (-0.447, 0.036) |  | -0.103 | -0.206 (-0.447, 0.035) |  | -0.111 |
|  | Ever preeclampsia , y/n | 0.231 ( 0.084, 0.379) | 0.224 | 0.206 | -0.011 (-0.177, 0.155) |  |  | 0.006 (-0.158, 0.171) |  |  |
| Lifestyle | Alcohol (drinks per month) | 0.006 ( 0.004, 0.008) | 0.006 | 0.006 | 0.005 ( 0.003, 0.007) | 0.005 | 0.003 | 0.003 ( 0.001, 0.006) | 0.004 | 0.003 |
|  | BMI kg/m2 | 0.002 (-0.004, 0.008) |  | 0.001 | -0.034 (-0.041, -0.028) | -0.034 | -0.034 | -0.038 (-0.044, -0.031) | -0.039 | -0.037 |
|  | Exercise hours per month | 0.001 (-0.002, 0.004) |  |  | 0.005 ( 0.001, 0.008) | 0.005 | 0.002 | 0.004 ( 0.000, 0.007) |  | 0.003 |
|  | Meat (1 oz. eq per week) | 0.016 ( 0.010, 0.021) | 0.016 | 0.015 | 0.004 (-0.002, 0.010) |  | 0.002 | 0.007 ( 0.001, 0.013) |  | 0.005 |
| Reproductive health | Estrogen or progesterone use (years) | -0.009 (-0.016, -0.002) |  | -0.005 | 0.002 (-0.005, 0.010) |  |  | 0.001 (-0.006, 0.009) |  |  |
|  | Postmenopause status (y/n) | 0.551 ( 0.447, 0.654) | 0.599 | 0.524 | 0.035 (-0.081, 0.152) |  |  | 0.045 (-0.073, 0.163) |  | 0.014 |
|  | Years pregnant and breastfeeding | -0.005 (-0.011, 0.001) |  | -0.004 | -0.001 (-0.008, 0.006) |  |  | -0.004 (-0.010, 0.003) |  | -0.001 |
|  | Years since LMP at interview | 0.015 ( 0.009, 0.021) | 0.015 | 0.014 | -0.002 (-0.009, 0.005) |  |  | -0.001 (-0.008, 0.006) |  |  |
| Supplement and Diet | Dietary + supplement calcium, dg | -0.009 (-0.015, -0.003) | -0.008 | -0.006 | 0.006 (-0.001, 0.013) |  | 0.000 | 0.001 (-0.006, 0.008) |  |  |
|  | Dietary + supplement iron, mg | 0.001 (-0.002, 0.003) |  |  | -0.002 (-0.004, 0.001) |  |  | 0.000 (-0.003, 0.002) |  |  |
| Performance | Apparent R^2^ | 0.308 | 0.271 | 0.276 | 0.082 | 0.073 | 0.078 | 0.110 | 0.090 | 0.101 |
|  | Calibration slope | 0.925 |  |  | 0.925 |  |  | 0.925 |  |  |
|  | Harrell corrected R^2^ | 0.290 | 0.261 | 0.270 | 0.067 | 0.062 | 0.077 | 0.086 | 0.078 | 0.099 |
